# Antimicrobial combination treatment including ciprofloxacin decreased the mortality rate of *Pseudomonas aeruginosa* bacteraemia: a retrospective cohort study

**DOI:** 10.1007/s10096-017-2907-x

**Published:** 2017-01-21

**Authors:** M. Paulsson, A. Granrot, J. Ahl, J. Tham, F. Resman, K. Riesbeck, F. Månsson

**Affiliations:** 1 Clinical Microbiology, Department of Translational Medicine, Lund University, Jan Waldenströms gata 59, 205 02 Malmö, Sweden; 2 Infectious Diseases Research Unit, Department of Translational Medicine, Lund University, Malmö, Sweden

## Abstract

Ineffective antimicrobial therapy of *Pseudomonas aeruginosa* bacteraemia increases mortality. Recent studies have proposed the use of antimicrobial combination therapy composed of a beta-lactam with either ciprofloxacin or tobramycin. To determine if combination therapy correlates to lower mortality and is superior compared to monotherapy, we investigated the effect of antimicrobial treatment regimens on 30-day mortality in a cohort with *Pseudomonas aeruginosa* bacteraemia. All cases of *P. aeruginosa* bacteraemia (*n* = 292) in southwest Skåne County, Sweden (years 2005–2010, adult population 361,112) and the whole county (2011–2012, 966,130) were identified. Available medical and microbiological records for persons aged 18 years or more were reviewed (*n* = 235). Antimicrobial therapy was defined as empiric at admission or definitive after culture results and was correlated to 30-day mortality in a multivariate regression model. The incidence and mortality rates were 8.0 per 100,000 adults and 22.9% (67/292), respectively. As expected, multiple comorbidities and high age were associated with mortality. Adequate empiric or definitive antipseudomonal treatment was associated with lower mortality than other antimicrobial alternatives (empiric *p* = 0.02, adj. *p* = 0.03; definitive *p* < 0.001, adj. *p* = 0.007). No difference in mortality was seen between empiric antipseudomonal monotherapy or empiric combination therapy. However, definitive combination therapy including ciprofloxacin correlated to lower mortality than monotherapy (*p* = 0.006, adj. *p* = 0.003), whereas combinations including tobramycin did not. Our results underline the importance of adequate antipseudomonal treatment. These data also suggest that *P. aeruginosa* bacteraemia should be treated with an antimicrobial combination including ciprofloxacin when susceptible.

**Electronic supplementary material:**

The online version of this article (doi:10.1007/s10096-017-2907-x) contains supplementary material, which is available to authorized users.

## Introduction

Bacteraemia caused by the opportunist *Pseudomonas aeruginosa* is a serious condition that has increased in incidence [1]. It is associated with high age, multiple comorbidities and advanced or prolonged healthcare [1]. Hence, the incidence rate of *P. aeruginosa* bacteraemia is likely to continue to rise as healthcare services are advancing and life expectancy continues to increase [1, 2]. It is one of the most common Gram-negative bloodstream infections (BSI), only preceded by *Escherichia coli* and *Klebsiella* spp. However, the mortality of *P. aeruginosa* BSI has consistently been reported to be higher than that of *E. coli* BSI (23–36%) [1, 3–8].

Treatment of pulmonary infections caused by *P. aeruginosa* has been the topic of several reviews [9–11]. Patients with cystic fibrosis (CF) are often colonised by *P. aeruginosa*, but it is rare that CF patients suffer from bacteraemia [12]. *Pseudomonas aeruginosa* also occasionally colonises chronic wounds and the gastrointestinal and urinary tracts, particularly in hospitalised patients or patients with indwelling catheters [13]. *Pseudomonas* strains causing long-term colonisation of CF patients have adapted to become less virulent, but are extremely resistant to antimicrobials due to altered efflux pumps, porins, beta-lactamases with extended substrate specificity and biofilm formation [14]. On the other hand, invasive isolates are generally more susceptible to antimicrobials, although strains with extensive antimicrobial resistance have been reported [15]. The optimal antimicrobial treatment regimens against *P. aeruginosa* in the airway may, thus, differ from optimal treatment regimens against bacteraemia and conclusions drawn from studies on pneumonia may not be generalisable to bacteraemia.

The most adequate treatment regimen of *P. aeruginosa* bacteraemia is a matter of debate. Despite the species being often highly resistant to antimicrobials, there are normally still several antimicrobial treatment regimens to choose from, either as monotherapy or combination therapy. Ineffective empiric antimicrobial therapy has been associated with increased mortality [16, 17]. Commonly used antipseudomonal drugs include penicillins with beta-lactamase inhibitors, certain cephalosporins, carbapenems, colistin, fluoroquinolones and aminoglycosides. Combination therapy is often administered to critically ill patients and most combinations include a beta-lactam antimicrobial together with either a fluoroquinolone or an aminoglycoside. Several studies have addressed the question as to whether to use monotherapy or combination therapy and the conclusions drawn are conflicting [16–20]. The assumption that combination therapy including either fluoroquinolones or aminoglycosides would have an equivalent effect on *P. aeruginosa* bacteraemia is, however, not necessarily correct. Earlier studies are inconclusive due to the insufficient number of patients in each group or stratification into combined groups of both fluoroquinolones and aminoglycosides. Hence, the effect on bacteraemia of adding a fluoroquinolone or an aminoglycoside to a beta-lactam is unclear at present.

We investigated the effect on 30-day mortality of different antimicrobial treatment regimens against *P. aeruginosa* bacteraemia. We observed that both empiric therapy on admission and definitive therapy after culture results affected mortality. Combination therapy with a beta-lactam and ciprofloxacin was significantly associated with a lower mortality compared to monotherapy. Moreover, the design of our population-based retrospective cohort allowed us to observe unbiased incidence rates and antimicrobial susceptibility patterns.

## Materials and methods

### Study population and setting

The cohort comprised the adult population (aged ≥18 years) of southwest Skåne County, Sweden during a 6-year period (2005–2010; adult population 361,112) in addition to the entire county during a period of 2 years (2011–2012; adult population 966,130). The area corresponded to the catchment area of the microbiological laboratory in Malmö that was expanded in 2011 because of fusion with an adjacent laboratory. The included laboratories analysed 100% of the blood cultures sampled in the area. Hospital care was provided by Skåne University Hospital and surrounding regional hospitals.

### Participants, study protocol and variables

**Fig. 1:**
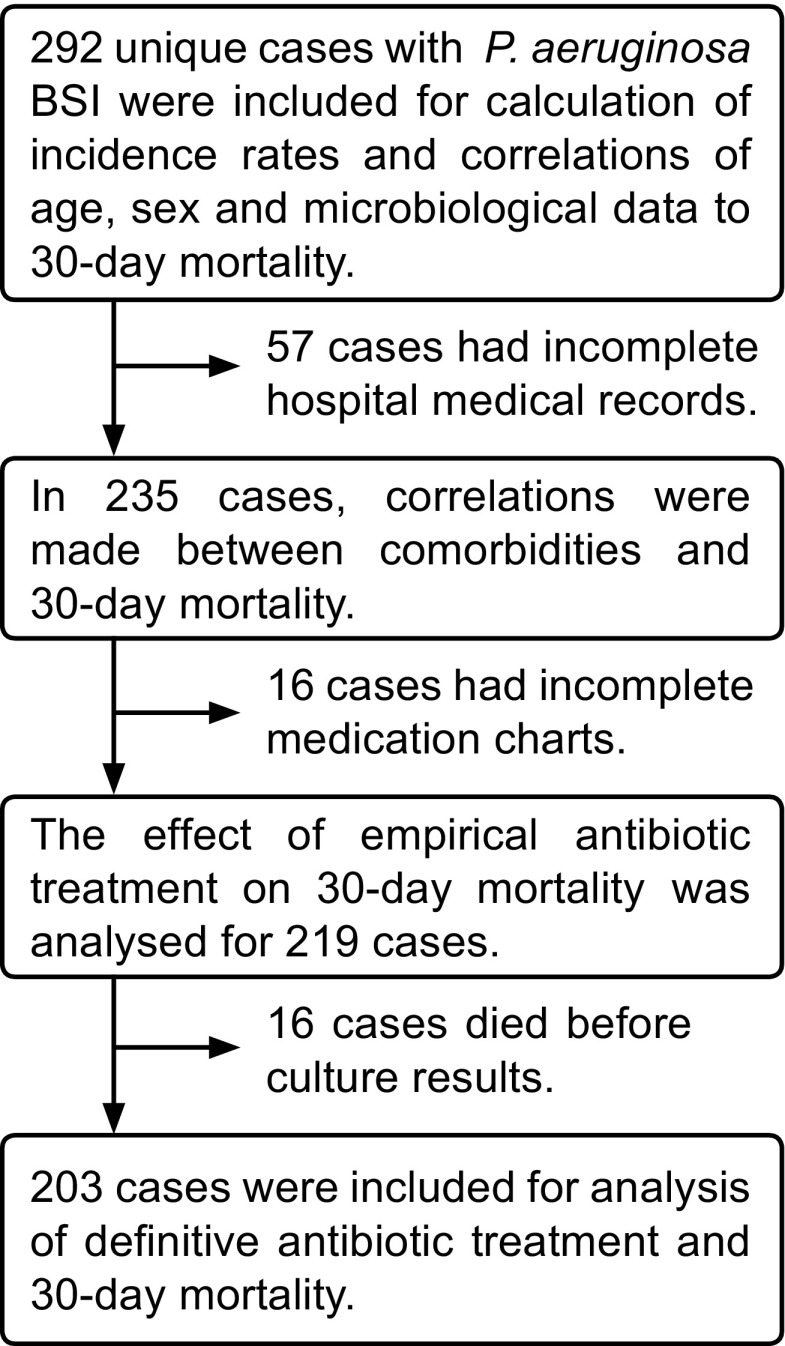
Patients included in this study. Flow chart summarising the number of cases in the present study and the reason for exclusion for some analyses

We identified 292 unique individuals with *P. aeruginosa* bacteraemia. Recurrent cases were only included once. Incidence rates were calculated using yearly population data collected from Statistics Sweden [21]. Microbiological culture data were collected from the laboratory’s records (wwLab; Autonik, Sköldinge, Sweden). Data on susceptibility and concurrent infections were collected, as were the time and date of preliminary and definitive culture results. Clinical data were collected from the hospital patient records (Melior; Siemens Healthcare Services, Upplands Väsby, Sweden). Thirty-day mortality was analysed as the outcome variable for all study cases (*n* = 292), as presented graphically in Fig. 1. Patients with available hospital medical records were included for correlations with comorbidities (*n* = 235). Sixteen patients had incomplete medication charts and were excluded from correlations with treatments (*n* = 219). Missing data were missing at random, with more missing records during the first years of the study, gradually decreasing over the study period. Randomness was controlled by comparing sex and age for missing and non-missing files. All recorded clinical variables and healthcare-related data are presented in the supplementary data, Table A1. The Charlson comorbidity index (CCI) was calculated to estimate the level of illness prior to the current bacteraemia (supplementary data, Table A2) [22]. Compound variables included pulmonary disease [chronic obstructive pulmonary disease (COPD), pulmonary fibrosis, asthma and cystic fibrosis] and heart disorder (congestive heart failure, ischaemic heart disease, cardiac arrhythmia and heart valve disease).

### Microbiological definitions and methods

*Pseudomonas aeruginosa* bacteraemia was defined as the isolation of the bacterial species in a blood culture collected from a patient at a hospital out-patient unit, a hospital ward or an emergency department using standard aseptic techniques. The term ‘bacteraemia’ was considered to be equivalent to BSI. All samples were cultured using the automated BacT/ALERT system (bioMérieux, Marcy l’Etoile, France). Isolates were identified by typical characteristics on agar plates and biochemical tests. Antimicrobial resistance was determined by disc diffusion on Mueller–Hinton agar plates, Etests (Biodisk, Solna, Sweden) or Vitek (bioMérieux) and subjected to antimicrobial susceptibility testing according to guidelines from the European Committee on Antimicrobial Susceptibility Testing (EUCAST) [23]. Physicians were notified when preliminary results on positive blood cultures were available.

### Antimicrobial treatment

Empiric therapy was defined as antimicrobial therapy administered from 8 h before sampling of the initial blood culture to the time the preliminary culture result was available. Antimicrobial treatment was classified as antipseudomonal if it included any of the following: ceftazidime, imipenem, meropenem, ciprofloxacin, piperacillin, colistin or combinations including gentamicin or tobramycin. Definitive therapy was defined as antimicrobial therapy given after preliminary or final culture results. Administered antimicrobials were compared with obtained antibiograms and classified as adequate if the bacterium was susceptible to the given drug in question.

### Statistical analysis

All data were entered into an Excel spreadsheet (Microsoft, Redmond, WA) and exported to SPSS Statistics version 23 (IBM, Armonk, NY) and Stata 14 (StataCorp, College Station, TX) for statistical analysis. Graphs were drawn in Prism 6 (GraphPad, La Jolla, CA). The results were expressed as median and interquartile range for continuous variables and as frequencies and percentages for categorical variables. Two-tailed *p*-values were calculated with Wilcoxon’s and Fisher’s exact test and values ≤0.05 were considered statistically significant. Odds ratios (ORs) were determined in the univariate analysis and presented with 95% confidence intervals (CIs). The adjusted OR for mortality was determined in a multivariate logistic regression model. We used stepwise backward selection to create the multivariable model in which all variables with *p*-values of ≤0.1 were entered, and variables with resulting *p*-values exceeding 0.2 were excluded. Age was stratified into categories, with 18–49 years as the index. A propensity score-adjusted analysis was performed that included the following covariates: age, sex, pulmonary, cardiovascular, renal, hepatic, neurological and malignant comorbidities, together with healthcare indicators: catheters, surgery, intensive care and coinfections. The propensity scores of receiving ciprofloxacin as combination therapy as well as monotherapy were each introduced into separate logistic regression models using ciprofloxacin treatment as a binary regression variable.

## Results

### The incidence of *P. aeruginosa* BSI increases with high age and male sex

**Fig. 2:**
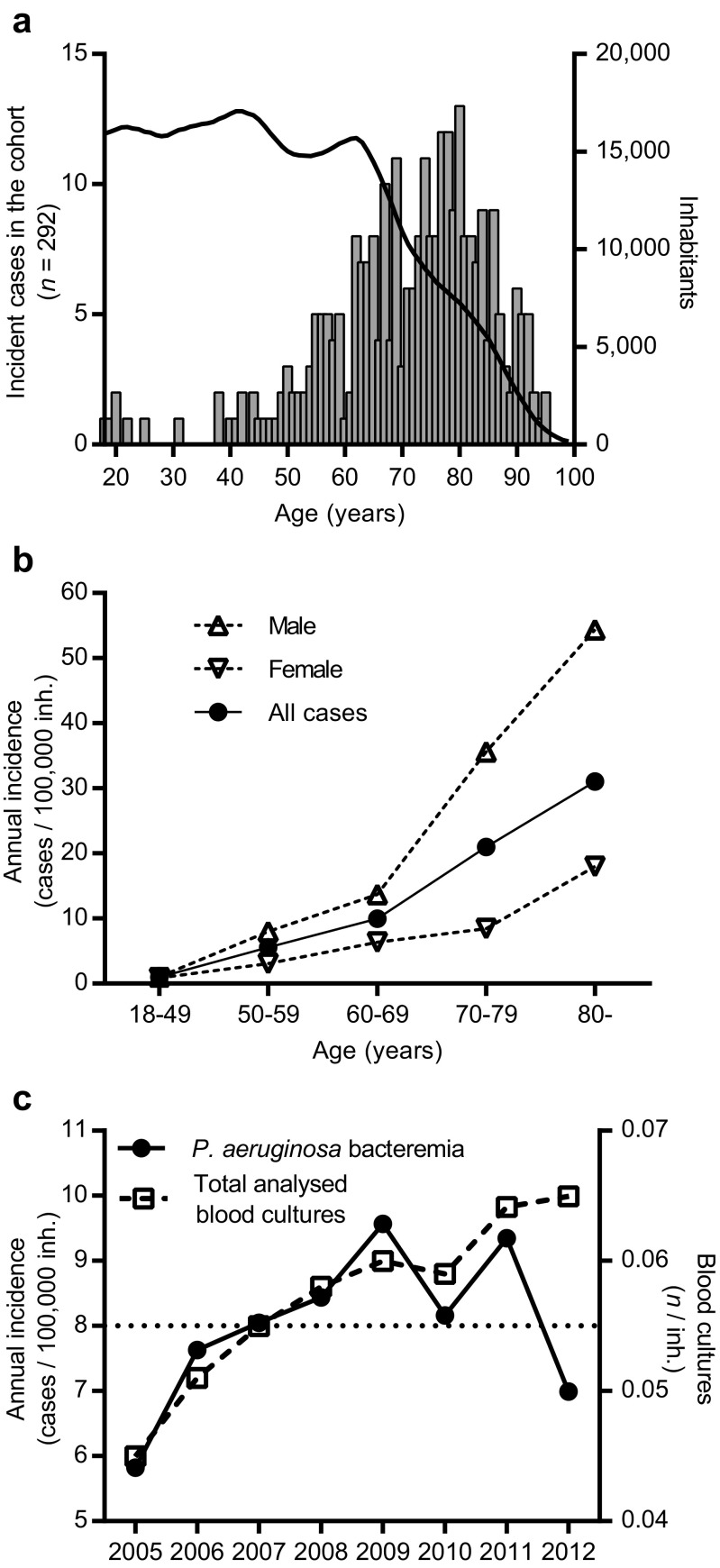
*Pseudomonas aeruginosa* bacteraemia incidence increases with age. Incident cases of *P. aeruginosa* bacteraemia were older (**a**, bars, left *y*-axis) than the population in Skåne County (**a**, curve, right *y*-axis). In all age groups, the incidence of *P. aeruginosa* bacteraemia was higher among males than females and increased with higher age (**b**). No increase in incidence was seen over the entire study period (**c**, left *y*-axis), even though the number of analysed blood cultures increased (**c**, right *y*-axis)

The average annual incidence rate of *P. aeruginosa* bacteraemia was 8.0 [standard deviation (SD) ± 1.22] cases per 100,000 adult inhabitants. The median age of the study cases was 74 years, well above the median age of the adult population (47 years, Fig. 2a) and the calculated incidence rate was highest for the oldest age groups (age ≥80 years: 31.0/100,000 adult inhabitants). As seen in previous studies, the incidence was higher for males than females (Fig. 2b) [5]. The most common infection focus was the urinary tract (94/235, 40.7%), followed by the respiratory tract (42/235, 18.2%), wounds (38/235, 16.5%) and intravenous catheters (9/235, 3.9%). The remaining individuals (48, 20.8%) had unknown infection site. Urinary tract focus was more common among men (males 77/159, 49.3% vs. females 17/75, 22.6%, *p* < 0.001) and most men with this focus had had a urinary catheter for more than one week (51/77, 66.2% vs. other foci 18/61, 22.8%, *p* < 0.001). During the first five years of the study, a yearly increase was seen in incident cases. This trend, however, ceased in 2010 and, in total, we could not observe any significant change in incidence rate, although the number of analysed blood cultures increased throughout the study period (Fig. 2c).

### Comorbidities, high age and respiratory tract focus correlate to increased mortality

The overall 30-day mortality for the study population was 22.9% (67/292). As expected, the surviving population was younger (mean age 69.8 vs. 74.4 years, *p* = 0.02). Basic demographic data, comorbidities and healthcare-related data of all included patients are presented in Table 1. To assess the effect of multiple comorbidities, we calculated the CCI from age and comorbidities. The CCI and selected variables were correlated to 30-day mortality in univariate and multivariate regression analysis (Table 2). Females had a higher mortality rate than males (adj. *p* = 0.006) and a higher CCI correlated strongly to higher 30-day mortality (survivors mean CCI 6.9 vs. non-survivors 8.5, *p* = 0.002). Pulmonary, haematological or metastatic malignant diseases were significantly associated with high 30-day mortality, whereas recent chemotherapy or diabetes mellitus correlated with lower 30-day mortality. Infection focus in the respiratory tract was associated with higher mortality and urinary tract infection or the presence of a urinary catheter with lower mortality. Coinfection with other pathogens was detected in 90 (31%) cases. The most common copathogens were *Enterobacteriaceae* but also a diverse flora of enterococci, staphylococci and anaerobes were recorded. Coinfections were associated with increased 30-day mortality (*p* = 0.02). Nosocomial infection defined as being a resident at a nursing home or being hospitalised for more than one week before blood culture was not associated with increased mortality rates.

**Table 1:** Basic demographic data of cases in this study

Characteristic	Male (*n* = 159)		Female (*n* = 76)	
Age	74 (63–80)		74 (63–83)	
Charlson score	7 (5–9)		6 (5–9)	
Pulmonary disease*	34 (21.4)		15 (19.7)	
COPD	17 (10.7)		7 (9.2)	
Cystic fibrosis	1 (0.6)		0 (0.0)	
Heart disorder*	62 (39.0)		25 (32.9)	
Peripheral vascular disease	40 (25.2)		15 (19.7)	
Vascular graft	11 (6.9)		4 (5.3)	
Diabetes mellitus	50 (31.4)		22 (28.9)	
Renal failure	36 (22.6)		6 (7.9)	
Chronic liver disease	4 (2.5)		4 (5.3)	
Neurological paresis	4 (2.5)		0 (0.0)	
Immunosuppression	35 (22.0)		17 (22.4)	
Chemotherapy in the last 6 months	30 (18.9)		22 (28.9)	
Solid malignancy	45 (28.3)		28 (36.8)	
Metastasis	24 (15.1)		15 (19.7)	
Haematological disease	26 (16.4)		10 (13.2)	
Neutropaenia	24 (15.1)		14 (18.4)	
AIDS	1 (0.6)		0 (0.0)	
Burn wounds	4 (2.5)		2 (2.6)	
Urinary catheter >1 week	70 (44.0)		18 (23.7)	
Hospitalised >1 week	58 (36.5)		24 (31.6)	
Surgery in the last month	34 (21.4)		14 (18.4)	
Resident at nursing home	26 (16.4)		7 (9.2)	

Age for all cases (*n* = 292) and characteristics for cases with full medical records (*n* = 235). Continuous variables are expressed as median (interquartile range) and categorical variables as observed numbers (percentage). Compound variables are marked with *

**Table 2:** Thirty-day mortality correlated to selected characteristics

Characteristic (*n* = 292)	*n*	Died (%)		OR (95% CI)				*p*-value	adj. OR (95% CI)				adj. *p*-value
Male sex	201	40 (19.9)		0.59 (0.33–1.04)				0.07	**0.35** (**0.17**–**0.74**)				**0.006**
Age 18–49 years	22	2 (9.1)		1.00									
Age 50–59 years	36	6 (16.7)		2.00 (0.37–10.92)				0.42	2.72 (0.39–19.08)				0.31
Age 60–69 years	62	14 (22.6)		2.92 (0.61–14.03)				0.18	3.21 (0.51–20.03)				0.21
Age 70–79 years	83	20 (24.1)		3.17 (0.68–14.78)				0.14	3.58 (0.58–22.17)				0.17
Age ≥80 years	89	25 (28.1)		3.91 (0.85–17.95)				0.08	**6.60** (**1.13**–**38.49**)				**0.04**
Comorbidity (*n* = 235)													
Pulmonary disease	49	18 (36.7)		2.58 (1.29–5.14)				0.01	**3.05** (**1.34**–**6.94**)				**0.008**
COPD	24	8 (33.3)		1.89 (0.76–4.69)				0.20	0.61 (0.16–2.37)				0.47
Cystic fibrosis	1	0 (0.0)											
Heart disorder	87	22 (25.3)		1.33 (0.71–2.49)				0.42	1.50 (0.64–3.51)				0.35
Peripheral vascular disease	55	13 (23.6)		1.14 (0.56–2.34)				0.71	2.26 (0.96–5.30)				0.06
Vascular graft	15	1 (6.7)		0.24 (0.03–1.83)				0.20	0.18 (0.02–1.69)				0.13
Diabetes mellitus	63	9 (14.3)		0.49 (0.22–1.08)				0.08	**0.46** (**0.22**–**0.96**)				**0.04**
Renal failure	42	10 (23.8)		1.12 (0.51–2.46)				0.84	1.96 (0.74–5.17)				0.18
Chronic liver disease	9	3 (33.3)		1.79 (0.43–7.40)				0.42	1.27 (0.22–7.21)				0.79
Neurological paresis	3	1 (33.3)		1.16 (0.12–11.36)				1.00	7.61 (0.45–128.36)				0.16
Immunosuppression	52	11 (21.2)		0.91 (0.43–1.93)				0.85	0.75 (0.26–2.18)				0.59
Chemotherapy in the last 6 months	52	12 (23.1)		1.05 (0.50–2.19)				1.00	**0.21** (**0.07**–**0.66**)				**0.007**
Solid malignancy	73	20 (27.4)		1.55 (0.81–2.94)				0.23	0.99 (0.37–2.68)				0.98
Metastasis	39	14 (35.9)		2.33 (1.11–4.90)				0.03	**7.12** (**2.32**–**21.79**)				**0.001**
Haematologic disease	42	11 (26.2)		1.63 (0.74–3.59)				0.28	**5.47** (**1.85**–**16.17**)				**0.002**
Neutropaenia	38	11 (28.9)		1.51 (0.69–3.30)				0.30	1.33 (0.41–4.28)				0.63
AIDS	1	0 (0.0)											
Burn wounds	6	2 (33.3)		1.77 (0.32–9.95)				0.62	4.08 (0.45–36.68)				0.21
Composite comorbidity score (*n* = 235)													
Charlson score ≤4	44	4 (9.1)		1.00									
Charlson score 5–8	119	27 (22.7)		2.93 (0.96–8.94)				0.06	2.91 (0.95–8.92)				0.06
Charlson score 9–12	49	13 (26.5)		3.61 (1.08–12.08)				0.04	**3.67** (**1.09**–**12.41**)				**0.04**
Charlson score ≥13	21	8 (38.1)		6.15 (1.59–23.82)				0.009	**7.05** (**1.79**–**27.86**)				**0.005**
Healthcare-related (*n* = 235)													
Urinary catheter >1 week	88	18 (20.5)		0.87 (0.45–1.65)				0.75	1.09 (0.50–2.39)				0.82
Surgery last month	48	9 (18.8)		0.76 (0.34–1.69)				0.56	0.98 (0.38–2.51)				0.96
Hospitalised >1 week	82	20 (24.4)		1.21 (0.64–2.29)				0.62	1.61 (0.76–3.41)				0.22
Resident at nursing home	33	8 (24.2)		1.15 (0.48–2.72)				0.82	1.16 (0.41–3.32)				0.78
Polymicrobial infection	90	22 (24.4)		1.13 (0.62–2.02)				0.68	**2.52** (**1.18**–**5.40**)				**0.02**
Origin of infection (*n* = 235)													
Urinary tract	82	12 (14.6)		0.37 (0.18–0.75)				0.005	**0.28** (**0.12**–**0.65**)				**0.003**
Respiratory tract	44	17 (38.6)		2.81 (1.38–5.70)				0.003	**2.81** (**1.13**–**7.01**)				**0.03**
Wound	38	12 (31.6)		1.81 (0.84–3.90)				0.13	1.99 (0.81–4.92)				0.13
Central venous catheter	9	0 (0.0)											
Other/unknown	50	11 (22.0)		0.99 (0.47–2.11)				0.98	1.16 (0.47–2.83)				0.75

Patient characteristics correlated to 30-day mortality and presented with odds ratio (*OR*), adjusted odds ratio (*adj. OR*) and 95% confidence interval (*95% CI*). The multivariable model contained age, sex, pulmonary disease, vascular graft, peripheral vascular disease, chemotherapy in the last 6 months, haematological diseases including malignancies, metastasis, diabetes mellitus and neurological paresis. Significant values are in **bold font**

### Antimicrobial resistance against *P. aeruginosa* did not increase during the study period

Antimicrobial resistance was generally low for the studied bacterial strains compared to previous studies. Imipenem resistance was 6.8%, meropenem 6.3%, piperacillin–tazobactam 6.2%, tobramycin 0.7%, ceftazidime 5.5% and ciprofloxacin 10.0%. Except for a peak in meropenem resistance in 2008, the antimicrobial resistance did not increase during the study period (supplementary data, Table A3). In univariate correlations, only meropenem resistance was associated with increased 30-day mortality (6 fatalities from 13 cases, 46.2%, *p* = 0.033).

### Empiric antimicrobial treatment and importance for mortality

Of the 219 patients with complete medication charts, almost all patients (93.2%) received antimicrobial treatment after cultures were drawn. In 47.5%, adequate empirical antimicrobial agents were given according to the antibiograms. The most common empiric treatment was cefotaxime (36.5%), followed by tobramycin (18.3%) and piperacillin (16.9%). The empiric use of any other antimicrobial was less than 10%. The administration of tobramycin was, in all cases, combined with another antimicrobial. Monotherapy was given to 125 patients (57.1%) and 79 patients received combination therapy (36.1%).

The choice of empiric treatment was associated with 30-day mortality in our cohort in direct correlations and when entered into a multivariable regression model (Table 3, upper section). Treatment with any effective antipseudomonal significantly decreased the 30-day mortality to 15.4% (adj. *p* = 0.03). In contrast, an increased 30-day mortality (53.3%; adj. *p* = 0.01) was observed when antimicrobials were not administered. Forty cases (18.2%) received empiric combination treatment including tobramycin, which did not decrease the mortality rate compared to monotherapy. Only five cases (2.3%) received empirical combination therapy including ciprofloxacin.

**Table 3:** The antimicrobial treatment choice influences 30-day mortality

	30-Day mortality, empirical treatment (*n* = 219)												
	*n*	Died (%)		OR (95%)				*p*-value	adj. OR (95% CI)				adj. *p*-value
Cefotaxime or cefuroxime	100	23 (23.0)		1.06 (0.56–2.00)				0.87	0.68 (0.31–1.49)				0.34
Benzylpenicillin	8	4 (50.0)		3.51 (0.85–14.42)				0.08	3.09 (0.52–18.38)				0.22
Imipenem or meropenem	34	5 (14.7)		0.55 (0.20–1.51)				0.25	0.84 (0.23–3.12)				0.79
Piperacillin–tazobactam	37	7 (18.9)		0.78 (0.32–1.90)				0.58	0.61 (0.20–1.89)				0.39
Ciprofloxacin	11	1 (9.1)		0.33 (0.04–2.64)				0.30	0.57 (0.06–5.56)				0.63
Combination including tobramycin	40	10 (25.0)		1.20 (0.54–2.66)				0.66	1.10 (0.39–3.11)				0.85
Any other combination	39	6 (15.4)		0.58 (0.23–1.48)				0.30	0.40 (0.13–1.27)				0.12
No empirical antibiotic treatment	15	8 (53.3)		4.52 (1.55–13.20)				0.007	**5.84** (**1.43**–**23.84**)				**0.01**
Adequate antipseudomonal treatment	104	16 (15.4)		0.45 (0.23–0.88)				0.02	**0.37** (**0.16**–**0.89**)				**0.03**
	30-Day mortality, definitive treatment (*n* = 203)												
	*n*	Died (%)		OR (95% CI)				*p*-value	adj. OR (95% CI)				adj. *p*-value
Cefotaxime or cefuroxime	10	4 (40.0)		3.93 (1.04–14.81)				0.043	5.59 (0.94–33.35)				0.06
Imipenem or meropenem	43	5 (11.6)		0.65 (0.23–1.80)				0.41	1.26 (0.35–4.49)				0.73
Piperacillin–tazobactam	67	11 (16.4)		1.05 (0.47–2.32)				1.00	1.07 (0.40–2.87)				0.89
Ceftazidime	21	1 (4.8)		0.24 (0.03–1.88)				0.18	0.19 (0.02–1.91)				0.16
Ciprofloxacin, monotherapy	25	2 (8.0)		0.43 (0.10–1.92)				0.27	0.32 (0.06–1.83)				0.20
Combination including ciprofloxacin	78	5 (6.4)		0.25 (0.09–0.68)				0.006	**0.16** (**0.05**–**0.55**)				**0.003**
Combination including tobramycin	35	4 (11.4)		0.65 (0.21–1.97)				0.44	1.23 (0.31–4.91)				0.77
Adequate antipseudomonal treatment	174	20 (11.5)		0.17 (0.07–0.41)				<0.001	**0.17** (**0.05**–**0.62**)				**0.007**

The effect of empiric and definitive antimicrobial treatment on 30-day mortality. Correlations to 30-day mortality presented as odds ratio (*OR*) with 95% confidence interval (*95% CI*) and *p*-values. The multivariable model contained age, sex, lung disease, vascular graft, peripheral vascular disease, chemotherapy in the last 6 months, metastasis, haematological disease, diabetes mellitus and neurological paresis, coinfections, treatment in the intensive care unit, tracheal intubation and urinary catheter. Significant adjusted *p*-values are in **bold**. Treatment regimens given to less than five patients are not shown in this table

### Definitive antimicrobial therapy with ciprofloxacin combinations increases survival

Sixteen patients (7.3%) died before any culture results were available. All surviving patients (*n* = 203) were administered definitive antimicrobial treatment following positive blood culture results with *P. aeruginosa*. The majority of cases (174; 85.7%) were given an effective antipseudomonal treatment. Preliminary culture results were provided for 93.1% and the mean time to preliminary culture result was 1.96 days (SD ± 1.19) and 3.68 days (SD ± 1.63) to final results. More than half were treated with ciprofloxacin (50.7%), as a part of a combination therapy (38.4%) or as monotherapy (12.3%). Piperacillin–tazobactam was given to 33.0%, carbapenem to 21.2%, tobramycin to 17.2% and, finally, ceftazidime to 10.3% of the cases. Other antimicrobial regimens were administered to less than 10% of patients.

The choice of an adequate definitive antipseudomonal treatment matching the antibiogram significantly decreased 30-day mortality (adj. *p* = 0.007) when analysed in our multivariable regression model (Table 3; lower section). Targeted monotherapy was not significantly associated with decreased 30-day mortality, whereas inadequate treatment with cefotaxime or cefuroxime as monotherapy increased mortality up to 40% (4/10).

**Fig. 3:**
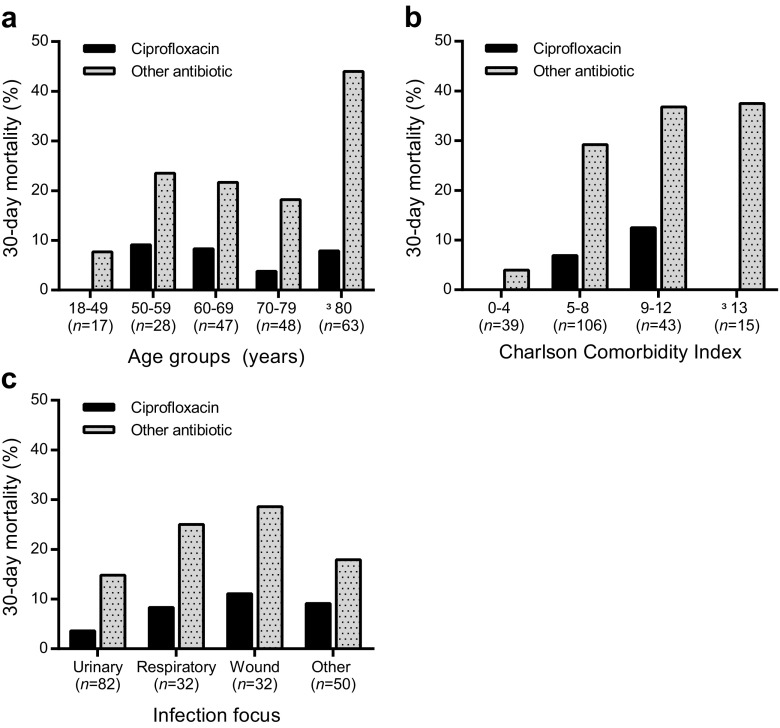
Ciprofloxacin-treated cases had a lower 30-day mortality. Thirty-day mortality rates in percent after treatment with ciprofloxacin or other antimicrobial drug as definitive therapy when culture results were available. The results were stratified by age groups (**a**), comorbidity as defined by the Charlson comorbidity index (CCI) (**b**) or infection focus (**c**)

In contrast to any individual monotherapy, definitive combination therapy with ciprofloxacin decreased the 30-day mortality (adj. *p* = 0.003), whereas combinations including tobramycin did not affect the mortality. To disclose involuntary selection bias in the group receiving ciprofloxacin, a propensity score was calculated. The effect of the addition of ciprofloxacin was independent of confounders (age, sex, comorbidities and intensive care treatment, as well as choice of antimicrobial agent). With the available covariates, we did not find any evidence of selection bias to any of the ciprofloxacin/no ciprofloxacin groups. Propensity score-adjusted analyses did not change the effect on mortality for ciprofloxacin combination therapy (propensity score-adjusted OR 0.16, 95% CI 0.05–0.53, *p* = 0.003 vs. OR 0.16, 95% CI 0.05–0.53, *p* = 0.003). For ciprofloxacin monotherapy, the association remained non-significant (propensity score-adjusted OR 0.59, 95% CI 0.04–9.9, *p* = 0.72 vs. OR 0.32, 95% CI 0.06–1.83, *p* = 0.20). The lower mortality associated with ciprofloxacin combination therapy was independent of age, CCI and origin of infection (Fig. 3a–c). Importantly also, patients in intensive care units that were treated with ciprofloxacin had a lower 30-day mortality than those receiving other antimicrobials (0 dead of 9 treated: 0% vs. other: 7/19, 36.8%, adj. *p* = 0.035).

## Discussion

In this retrospective cohort study, we provide evidence that the choice of antimicrobial treatment affected the 30-day mortality in patients suffering from *P. aeruginosa* bacteraemia. Despite the average time to culture results being as short as 48 h, inadequate empiric antimicrobial treatment on admission negatively affected mortality rates. Similarly, inadequate definitive treatment after blood culture results was also associated with higher mortality rates. Particularly, definitive combination treatment that included ciprofloxacin favourably affected the 30-day mortality.

In total, we identified 292 patients with *P. aeruginosa* bacteraemia between 2005 and 2012. The overall 30-day mortality in this study was 22.9% and the annual incidence was 8 per 100,000 inhabitants, with higher rates among men and with increasing age. This was in line with previous studies, which have reported 30-day mortality rates ranging from 23% to 36.5% and incidence rates in the range 3.6–10.8 cases per 100,000 inhabitants and year [1, 3–8]. The incidence was higher for males than females, which was accounted for by the higher incidence among males with urinary catheter. Although the number of blood cultures steadily increased during the study period, no increase in the incidence of *P. aeruginosa* bacteraemia was seen over the entire study period. However, in parallel to the increase reported in the UK, the incidence rate in our study rose between 2005 and 2009 [1]. The explanation for the rising incidence during the first several years of the study and the concurrent increase in the UK is, at present, unclear and no epidemiological link is known.

The importance of correct empiric therapy on admission has been debated and, in the present study, the initial antimicrobial choice was important, as patients who received adequate antipseudomonal treatment had significantly lower mortality rates [3, 17, 24]. No individual empirical antimicrobial monotherapy or combination therapy was associated with changes in mortality. The large number of different administered antimicrobials resulted in low statistical power for all but the most commonly used drugs and only five patients received empirical ciprofloxacin combination treatment.

After positive blood cultures with *P. aeruginosa*, adequate definitive antipseudomonal treatment decreased the 30-day mortality. In contrast to ciprofloxacin combinations, combination therapy including tobramycin was not superior to monotherapy, similarly to that previously reported [20]. To find the potential influence of selection bias on our results, a propensity score was calculated to ensure comparability of the cohort receiving ciprofloxacin and those receiving other treatments, but no such bias could be identified. A few previous studies have investigated whether combination therapy was favourable against *P. aeruginosa* bacteraemia. For example, Kim et al. showed that adequate combination therapy of any sort was beneficial for a subgroup of bacteraemic patients with febrile neutropaenia [18]. Peña et al. reported, however, that the choice between combination therapy and single-drug therapy did not affect outcome [17]. Furthermore, in a recent meta-analysis focusing on empiric treatment, no difference in mortality was seen between the study groups, but in contrast to the present investigation, no analysis was made separately for aminoglycoside and ciprofloxacin combination therapy [19]. Our results are supported by DiMondi et al., who investigated the short-term outcome of bacteraemia and pneumonia; ciprofloxacin combinations comprised 90% of the combination therapies that were associated with favourable outcome [16]. Both beta-lactams and ciprofloxacin are highly efficient against *P. aeruginosa* in vitro. The reason for the favourable outcome for ciprofloxacin combination-treated patients in the present study is unknown. Combination therapy broadens the antimicrobial spectrum and synergy between antimicrobial drugs has been described, but synergy between ciprofloxacin and beta-lactams has not been reported [25].

In our cohort, 10.0% of the bacterial strains had reduced susceptibility for ciprofloxacin. This should be compared to three BSI studies from the USA that reported varying resistance levels (range 4.7–31%), with the highest levels from Maryland, where a majority of cases had been hospitalised for more than three days at inclusion [3, 4, 24]. Local knowledge of resistance levels is important, but it may be difficult to derive current resistance levels from surveillance programmes, as BSI may not be reported and, often, bacteraemia-causing bacteria are more susceptible. For comparison, the fluoroquinolone resistance in Skåne County of *P. aeruginosa* bacteraemia strains was 9.8% in 2011, whereas in the same year, 88.1% of *P. aeruginosa* strains from the respiratory tract of patients with CF were resistant to fluoroquinolones (unpublished data). Hence, combinations of antimicrobials including ciprofloxacin may be the most effective antipseudomonal treatment against bacteraemia, but the results are not, however, generalisable to pneumonia.

Patients suffering from BSI were of higher age than the general population and had multiple comorbidities, which also correlated to higher 30-day mortality, as did pulmonary disease (and infectious foci in the respiratory tract), malignant disease with metastasis and haematological malignancies. As inadequate antimicrobial therapy increased mortality, the likelihood of *P. aeruginosa* BSI should be considered at the choice of empiric antimicrobial therapy. Not all septic patients need empiric antipseudomonal treatment at admission, but it could be considered for patients with increased risk of *Pseudomonas* bacteraemia and with high risk of mortality. In addition to critically ill patients, these results suggest that patients with neoplasia and the elderly with multiple comorbidities could be eligible for such treatment. However, laboratory tests in vitro have shown that the use of ciprofloxacin at sub-MIC concentrations lead to bacterial mutations, causing resistance against both ciprofloxacin and beta-lactam antimicrobials [26]. Hence, any decisions to use ciprofloxacin should be made wisely in order to limit further resistance development and monotherapy with ciprofloxacin should probably be avoided.

Our study cohort had a minimised population selection bias and, consequently, allowed for observation of, in total, 4.2 million adult person-years. The records at the microbiology laboratories allowed us to identify every occurrence of *P. aeruginosa* bacteraemia. This population-based approach allowed for a valid analysis of the incidence rate. We were not able to use a scoring system for severity of disease; instead this was determined by treatment at an intensive care unit. In the future, a prospective randomised interventional study to examine the effects of treatment regimens would be of high value, although the relative infrequency of *P. aeruginosa* bacteraemia would make this challenging in practice. An alternative would be a larger retrospective trial with emphasis on markers of acute severe disease.

In conclusion, this study gives an indication that *P. aeruginosa* bacteraemia should be treated with definitive antimicrobial drug combination regimens including ciprofloxacin when susceptible. Inadequate empiric antipseudomonal treatment on admission or inadequate definitive therapy after notification of positive blood cultures was associated with increased mortality. These results are of particular importance to those at the greatest risk for *P. aeruginosa* bacteraemia, the elderly patients with multiple comorbidities and patients with malignancy. Appropriate antipseudomonal treatment should be considered for these patients as early as possible to minimise the risk of death.

## Electronic supplementary material

Below are the links to the electronic supplementary material.

Table A1 Recorded clinical and healthcare-related variables. Variables marked with * were combined in ‘Pulmonary disease’ and those with ** in ‘Heart disorder’. (DOCX 18 kb)

Table A2 Charlson comorbidity index (CCI). Variables and scoring used to calculate the CCI in the current study [22]. (DOCX 15 kb)

Table A3 Reported resistance rates in percent for the most commonly used antimicrobial drugs in this study. Imipenem (*n* = 292), meropenem (*n* = 204), piperacillin (*n* = 292), tobramycin (*n* = 281), ceftazidime (*n* = 290) and ciprofloxacin (*n* = 291). (DOCX 15 kb)

## Abbreviations

AIDS: Acquired immune deficiency syndrome
BSI: Bloodstream infection
CCI: Charlson comorbidity index
CF: Cystic fibrosis
CI: Confidence interval
COPD: Chronic obstructive pulmonary disease
MIC: Minimum inhibitory concentration
OR: Odds ratio
SD: Standard deviation
