# Supplementary material for: Antimicrobial combination treatment including ciprofloxacin decreased the mortality rate of Pseudomonas aeruginosa bacteraemia: a retrospective cohort study
Source: Eur J Clin Microbiol Infect Dis. 2017 Jan 21;36(7):1187–96. doi: 10.1007/s10096-017-2907-x (PMC5495847; doi:10.1007/s10096-017-2907-x)
Supplement: Supplementary file 1 — Recorded clinical and healthcare-related variables. Variables marked with * were combined in ‘Pulmonary disease’ and those with ** in ‘Heart disorder’. (DOCX 18 kb) [file 10096_2017_2907_MOESM1_ESM.docx]

| **Comorbidities** |
| --- |
| Chronic pulmonary disease |
| Chronic obstructive pulmonary disease* |
| Pulmonary fibrosis* |
| Asthma* |
| Cystic fibrosis* |
| Acute myocardial infarction |
| Ischemic heart disease** |
| Congestive heart failure** |
| Cardiac arrythmia** |
| Heart valve disease** |
| Peripheral vascular disease |
| Vascular grafts |
| Cerebrovascular disease |
| Dementia |
| Connective tissue disease |
| Ulcer disease |
| Diabetes mellitus without complications |
| Diabetes mellitus with end-organ damage |
| Moderate or severe renal disease |
| Mild liver disease |
| Moderate or severe liver disease |
| Burn wounds |
| Hemiplegia |
| Immunosuppression |
| Chemotherapy last 6 months |
| Any tumour |
| Metastatic solid tumour |
| Leukaemia |
| Lymphoma |
| Other haematologic disease |
| Neutropenia |
| AIDS |
|  |
| **Health care related variables** |
| Urinary catheter |
| Recent hospital admissions |
| Recent surgery |
| Central venous catheter |
| Tracheal intubation |
| Nursing home |
