# Supplementary material for: Antimicrobial combination treatment including ciprofloxacin decreased the mortality rate of Pseudomonas aeruginosa bacteraemia: a retrospective cohort study
Source: Eur J Clin Microbiol Infect Dis. 2017 Jan 21;36(7):1187–96. doi: 10.1007/s10096-017-2907-x (PMC5495847; doi:10.1007/s10096-017-2907-x)
Supplement: Supplementary file 2 — Charlson comorbidity index (CCI). Variables and scoring used to calculate the CCI in the current study [22]. (DOCX 15 kb) [file 10096_2017_2907_MOESM2_ESM.docx]

| **Charlson Comorbidity Index** |  |
| --- | --- |
| Variable | Score |
| Acute myocardial infarction | 1 |
| Congestive heart failure | 1 |
| Peripheral vascular disease | 1 |
| Cerebrovascular disease | 1 |
| Dementia | 1 |
| Chronic pulmonary disease | 1 |
| Connective tissue disease | 1 |
| Ulcer disease | 1 |
| Mild liver disease | 1 |
| Diabetes mellitus without complications | 1 |
| Diabetes mellitus with end-organ damage | 2 |
| Hemiplegia | 2 |
| Moderate or severe renal disease | 2 |
| Any tumour | 2 |
| Leukaemia | 2 |
| Lymphoma | 2 |
| Moderate or severe liver disease | 3 |
| Metastatic solid tumour | 6 |
| Aids | 6 |
| *In addition, age was included in the index as follows:* | |
| 50–59 years | 1 |
| 60–69 years | 2 |
| 70-79 years | 3 |
| 80-89 years | 4 |
| 90-99 years | 5 |
