# Supplementary material for: Antimicrobial combination treatment including ciprofloxacin decreased the mortality rate of Pseudomonas aeruginosa bacteraemia: a retrospective cohort study
Source: Eur J Clin Microbiol Infect Dis. 2017 Jan 21;36(7):1187–96. doi: 10.1007/s10096-017-2907-x (PMC5495847; doi:10.1007/s10096-017-2907-x)
Supplement: Supplementary file 3 — Reported resistance rates in percent for the most commonly used antimicrobial drugs in this study. Imipenem (n = 292), meropenem (n = 204), piperacillin (n = 292), tobramycin (n = 281), ceftazidime (n = 290) and ciprofloxacin (n = 291). (DOCX 15 kb) [file 10096_2017_2907_MOESM3_ESM.docx]

|  | 2005 | 2006 | 2007 | 2008 | 2009 | 2010 | 2011 | 2012 | All years |
| --- | --- | --- | --- | --- | --- | --- | --- | --- | --- |
| Imipenem | 14.3% | 14.3% | 0.0% | 12.5% | 5.4% | 3.1% | 6.6% | 3.9% | 6.8% |
| Meropenem | 10.5% | 0.0% | 0.0% | 22.2% | 6.7% | 3.1% | 6.9% | 3.9% | 6.4% |
| Piperacillin-tazobactam | 0.0% | 7.1% | 6.7% | 9.4% | 8.1% | 3.1% | 6.6% | 5.9% | 6.2% |
| Tobramycin | 0.0% | 0.0% | 0.0% | 0.0% | 2.7% | 0.0% | 0.0% | 2.0% | 0.7% |
| Ceftazidime | 4.8% | 3.6% | 6.9% | 12.5% | 8.1% | 0.0% | 4.9% | 3.9% | 5.5% |
| Ciprofloxacin | 9.5% | 14.3% | 10.3% | 6.3% | 13.5% | 12.5% | 9.8% | 5.9% | 10.0% |
